# Prognostic Value of Melanoma Inhibitory Activity Protein in Localized Cutaneous Malignant Melanoma

**DOI:** 10.1155/2014/843214

**Published:** 2014-06-22

**Authors:** Angela Sandru, Eugenia Panaitescu, Silviu Voinea, Madalina Bolovan, Adina Stanciu, Sabin Cinca, Alexandru Blidaru

**Affiliations:** ^1^Department of Surgical Oncology, “Carol Davila” University of Medicine and Pharmacy, “Alexandru Trestioreanu” Oncologic Institute, Fundeni Street, No. 252, Sector 2, 022328 Bucharest, Romania; ^2^Department of Medical Informatics and Biostatistics, “Carol Davila” University of Medicine and Pharmacy, Bucharest, Romania; ^3^Department of Carcinogenesis and Molecular Biology, “Alexandru Trestioreanu” Oncologic Institute, Bucharest, Romania

## Abstract

*Background*. Cutaneous malignant melanoma (CMM) is a heterogeneous disease, acknowledged for its lack of predictability regarding clinical evolution. In order to appreciate a patient's individual prognosis, an attempt is made to find new tumor markers that parallel the disease progression. *Objective*. To identify if melanoma inhibitory activity (MIA) protein could represent a tool for selecting high risk early stages melanoma patients. *Method*. Between 2008 and 2013, 155 patients with CMM were treated in our clinic. 84 of them were classified into stages I and II, according to TNM 2009. MIA serum concentration was measured in all patients and 50 healthy donors. A cut-off value of 9.4 ng/ml was established using the ROC curve. *Results*. All patients were followed up by periodic investigations every 6 months. We have noticed that 66% of patients with MIA serum values at diagnosis greater than 9.4 ng/mL have relapsed, while only 5% of patients with MIA serum concentration below the estimated threshold, recurred during the follow-up period (*P* = 0.000). The death risk was 12 times higher in pathological MIA group of patients (*P* = 0.0001). *Conclusions*. Our data suggest that MIA is an independent prognostic factor for patients with localized CMM.

## 1. Introduction

Malignant melanoma represents the cutaneous neoplasia with the highest metastatic potential and a relatively unpredictable evolution, even in early stages. Fortunately, however, the detection of the disease in a localized phase allows it to be cured in most cases [1]. 

Statistics show that about 30% of people diagnosed with cutaneous malignant melanoma (CMM) develop metastases [2]. In these circumstances, finding new tools to identify those patients who although classified in stage I or II, so theoretically with a favorable outcome, still have a high risk of dissemination, represents a priority. A possibility in this regard might be the discovery of tumor markers whose expression in the blood stream would mirror the disease development.

Dozens of substances have been described which were deemed to have prognostic capacity in different stages of CMM. This presumed prognostic ability was suggested by the parallelism between serum concentration of these markers and body tumor burden that was noted in a significant percentage of cases. Nevertheless, only two of them are widely accepted: serum lactate dehydrogenase (LDH) and protein S100 [3, 4].

In recent years, researches in the field of autocrine growth-regulatory factors have led to the discovery of a new serum tumor marker, melanoma inhibitory activity (MIA), a protein synthesized by the malignant melanocytes and actively secreted in the extracellular compartment [5], from where a small amount reaches the bloodstream.

MIA has been isolated from the supernatant of malignant melanocytes cultures and initially it was thought to be a tumor suppressor factor, because, at this level, MIA was inhibiting the melanocytes proliferation by preventing their attachment to the culture plate [5, 6]. However, in vivo, MIA has a totally opposed action: its massive expression by malignant melanic cells but not by the normal ones enables the malignant melanocytes to detach from the extracellular matrix components, thus increasing their mobility and implicitly their metastatic potential [5, 6]. 

MIA protein interacts with fibronectin, laminin, and tenascin and masks their binding sites with integrins situated on the melanocytes membrane [5, 6]. In this way, MIA induces a decrease by 30–50% of the attachments between malignant melanocytes and the adjacent conjunctive tissue [7]. Consequently, a high serum MIA concentration seems to indicate the presence of an aggressive tumor clone and the existence of a metastatic tumor phenotype.

Starting from these data, the present study intends to prospectively determine the clinical significance of MIA serum concentration in patients with localized CMM, in an attempt to answer the following questions.

- Is there a link between MIA serum concentration and survival of patients with early stage CMM?
- Is a MIA value above the upper normal limit in a patient without evidence of disease a negative prognostic factor which should be taken into account in establishing the subsequent treatment and follow-up?

## 2. Material and Method

### 2.1. Group of Patients

Between June 2008 and May 2012, in Clinic II of Surgical Oncology of “Alexandru Trestioreanu” Oncologic Institute in Bucharest (IOB), 155 patients were diagnosed with CMM in various stages of disease and subsequently treated. Diagnosis of CMM was established, in most cases (153), by excisional biopsy of the primary tumor and patients were staged according to AJCC 2009. In order to characterize the status of the regional lymph nodes, lymphatic mapping and sentinel node biopsy were done for 61 patients with clinically and echographically normal regional lymphatic basins. The sentinel node was positive for malignant cells in only 10 cases.

For accurate staging, all patients were investigated by chest X-ray, abdominal and regional lymphatic basin ultrasound, and CT in suspected cases. After analyzing the obtained results, 84 of the subjects included in this study were classified in stages I and II. After surgery, they were submitted to periodic controls, every 6 months, which consisted in a complete physical exam, abdominal and regional lymphatic basin ultrasound, and, once a year, chest radiography.

### 2.2. MIA Samples

A number of 200 blood samples were obtained from 155 patients with CMM and 50 samples were obtained from healthy volunteers. Beforehand, patients were informed that they could participate in a clinical study and, in case of a positive response, a written approval was asked, in order to determine two blood tumor markers: MIA and S100 proteins (data referring to S100 are not presented in this paper).

After informed consent was obtained, 2 tubes of 5 mL blood were collected from each patient. For MIA samples, tubes with anticoagulant (EDTA) were used and the obtained plasma was kept at a temperature of −80°C for a variable period of maximum 12 months. Lipemic or strongly hemolyzed samples were eliminated from the work group. The samples processing was done in several batches within the IOB research laboratory. MIA serum value was measured with an ELISA type quantitative reaction of immunodetection by photometry, using a kit purchased from ROCHE Diagnostics, Roche Applied Science, Mannheim, Germany.

Regarding patients in stages I and II, MIA serum concentration was measured before surgery (excisional biopsy of the primary tumor or sentinel node biopsy). For some of the patients, multiple sampling was acquired during the postsurgical follow-up.

### 2.3. Statistical Analysis

For the statistical analysis the SPSS 15.0 system was used. For MIA values the receiver operating characteristics (ROC) and the area under the curve (AUC) were assessed. Survival time differences for the different MIA cut-offs were analyzed using the log rank test. Cox regression analyses were performed for the MIA cut-off value. As further multivariate method logistic regression was used, a *P* value of less than 0.05 was considered statistically significant.

## 3. Results

Of the 84 patients with localized disease, 54 were women and 30 men (Table 1). The mean age at diagnosis was 52.2 years. Primary tumor distribution varied with patients' sex: the most frequent localization on men was at the trunk level (46.7%) and on women at limbs level (64.8%). The recorded differences are very close to statistical significance (*P* = 0.079). The mean follow-up interval was 34.3 months, with a median of 32.8 months (the minimum surveillance period was 6.1 months).

**Figure 1:**
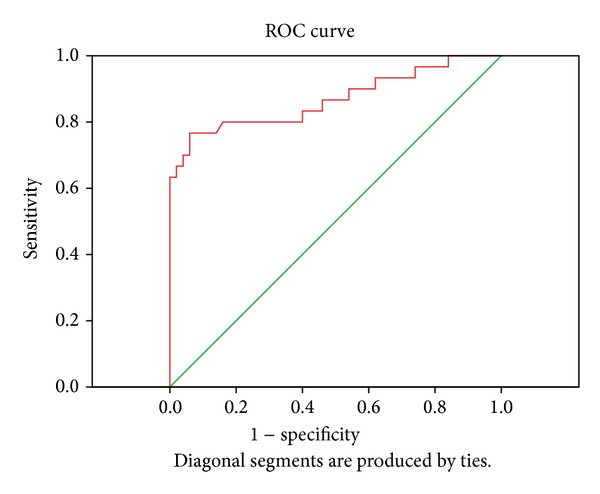
ROC curve for stage IV patients MIA values (AUC = 0.869).

Taking into account all MIA values (50 healthy donors and 155 patients with CMM), it was estimated, by using the ROC curve, that MIA cut-off value is 9.4 ng/mL. This cut-off point was calculated so that the sum of sensibility (76.7%) and specificity (94%) was maximum for patients with stage IV disease (Figure 1).

**Figure 2:**
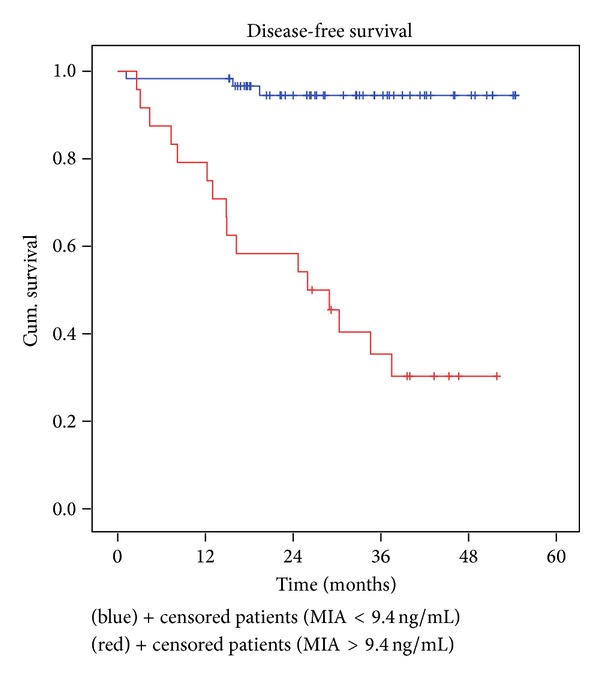
Kaplan-Meier disease-free survival for all stages I and II patients depending on MIA serum concentration. Red line: MIA serum concentration ≥ 9.4 ng/mL; blue line: MIA serum concentration < 9.4 ng/mL; *P* = 0.000.

In the group of stages I and II patients, 24 of them (~one-third) had a MIA serum concentration greater than 9.4 ng/mL preoperatively (Table 2). It is significant that 66.7% of these patients with elevated MIA values, that is, 16, developed regional or distance relapses, during the observation period. On the other hand, the disease evolved in only 5.0% of patients with MIA serum concentration within normal limits at diagnosis. Using univariate Cox analysis, we drew the conclusion that patients in stage I or II, with pathological MIA value before surgery, have a relapse rate 16 times higher than those with MIA within normal limits (hazard ratio = 16.11 with 95% CI = [4.68, 55.40]), which was translated in a significantly lower disease-free survival rate (*P* = 0.000) (Figure 2).

**Figure 3:**
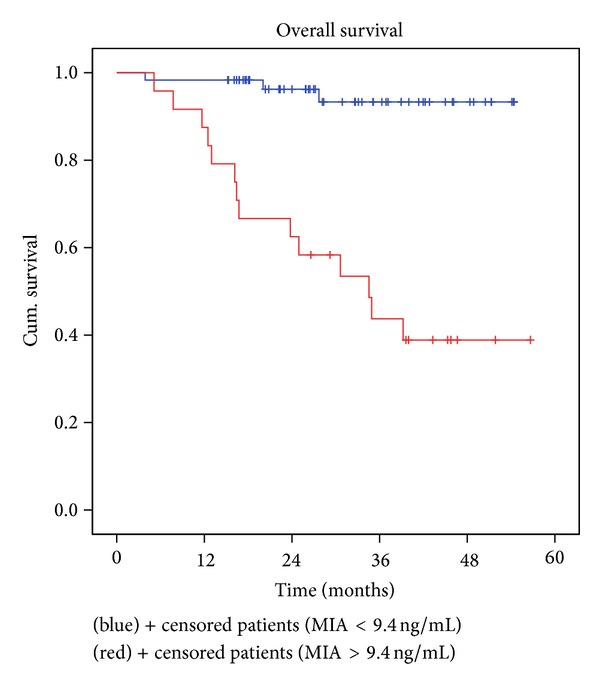
Kaplan-Meier overall survival for all stages I and II melanoma patients according to MIA serum concentration. Blue line: MIA serum concentration < 9.4 ng/mL; red line: MIA serum concentration ≥ 9.4 ng/mL; *P* = 0.000.

During the follow-up interval, 17 deaths were recorded in the group of patients enrolled in stages I and II; in other words, 20% of patients with localized disease died within 5 years of diagnosis (Table 3). The percentage is quite high compared with literature data and alarming at the same time. Trying to find an explanation for these unexpected and unpleasant results, we noted that, in 14 of the patients who died, the MIA serum concentration at diagnosis exceeded the threshold of 9.4 ng/mL, the highest measured value being 67.7 ng/mL. By analyzing the obtained data, we calculated that patients with localized CMM but with a MIA blood concentration greater than 9.4 ng/mL have 12 times higher death risk (hazard ratio = 12.30 with 95% CI = [3.53, 42.86]) compared to patients whose tumors do not overexpress MIA (*P* = 0.0001) (Figure 3).

Multivariate logistic regression was used to demonstrate that MIA serum concentration is an independent prognostic factor for survival, irrespective of TNM stage. For melanoma patients in the same stage of disease (stage I or II in this study), a MIA value greater than 9.4 ng/mL increases the death risk 33 times (*P* = 0.0000) (Table 4).

## 4. Discussions

The role of tumor markers in the initial stages of a neoplasia, regardless of its location, is still a subject of debate. As for CMM, both LDH and S100 have proven their utility in monitoring patients with metastasis [8] but have no role in the early stages of the disease. Actually, till now, no serum biomarker which might be able to suggest the evolution of patients with localized CMM has been identified [9].

In the complex network of autocrine and paracrine growth-regulatory factors involved in the progression of CMM, MIA seems to have a key role in the increase of malignant melanocytes motility and consequently in the initiation of the metastatic process. Therefore the hypothesis that an increased level of MIA protein in the blood of patients with localized CMM is associated with an increased risk of disease relapse seems plausible.

There is no unanimously accepted threshold for the upper limit of MIA serum concentration, as this varies between 6.5 ng/mL [10] and 18.7 ng/mL [11] in the literature. Within these limits each research team has set its own cut-off value depending on the number of investigated healthy volunteers, kits manufacturer's recommendations, and the calculation method applied. The most frequent upper limits used were 8.8 ng/mL [12], 10 ng/mL [13], 12 ng/mL [14], 14 ng/mL [15], and 15 ng/mL [12].

For the entire group studied by us, 155 patients with CMM and 50 healthy donors, we have established, using the ROC curve, a cut-off value of 9.4 ng/mL (data are not presented in this paper). Relating to this figure, we have assessed the recurrence and death risk for stages I and II patients from our group depending on diagnostic MIA serum concentration.

The data obtained suggests that there is a connection between the survival of patients with localized CMM and MIA serum concentration, a finding also presented in other papers [12, 13]. The 4-year disease-free survival was triple in the group of patients with MIA serum concentration in the normal range (Table 2, Figure 1), which is a further proof for MIA significant involvement in the processes of invasion and metastasis (*P* = 0.000).

Overall survival shows the same trend. Patients with CMM and MIA serum concentration at diagnosis lower than 9.4 ng/mL had a 4-year overall survival rate of 93.3% (Table 3, Figure 2), similar percentage to the literature data and significantly higher than that noted in patients with pathological MIA, 38.9% (*P* = 0.0000).

Multivariate analysis of the entire group of patients with CMM (155 patients in stages I–IV) revealed that MIA serum concentration represents an independent predictor for survival (*P* = 0.0109), together with the already acknowledged prognostic factors: clinical stage at diagnosis, sex of the patient, and lymphovascular invasion (data not shown in this paper).

## 5. Conclusions

A preoperative MIA serum concentration of 9.4 ng/mL or more in patients with localized CMM represents an alarm signal! In our study, elevated MIA values in patients with stages I and II CMM, properly staged, including sentinel node biopsy, have been associated with significantly lower DFS and OS. Therefore we consider that these patients should be monitored at shorter time intervals and thoroughly investigated in more detail in order to identify potential metastases.

Routine preoperative determination of MIA allows the selection of a group of patients with higher risk of relapse that might benefit from an adjuvant systemic treatment or participate in clinical trials. In our opinion MIA represents a useful tumor marker in both stratifying and adjusting the postoperative treatment and choosing the optimal follow-up for patients with localized CMM.

## Figures and Tables

**Table 1:** Patient demographics (*N* = 84 patients).

Tumor characteristics	Stage I	Stage II
Number of patients	28 (33.3%)	56 (66.7%)
Mean age (years)	51.07 (±10.6)	53.40 (±13.5)
Sex		
Female (%)	17 (60.7%)	37 (66.1%)
Male (%)	11 (39.3%)	19 (33.9%)
Mean Breslow depth (mm) Minimum/maximum	1.42 (±1.1) 0.22 mm	3.29 (±1.7) 12 mm
Localization		
Head/neck (%)	3 (10.7%)	4 (7.1%)
Trunk (%)	12 (42.9%)	35 (62.5%)
Extremity (%)	13 (46.4%)	17 (30.4%)

**Table 2:** Disease-free survival (DFS) rate of stages I and II melanoma patients according to MIA serum concentration.

MIA ng/mL	Number of patients	Number of relapses	1-year DFS	2-year DFS	3-year DFS	4-year DFS	Mean DFS (95% CI)	Median DFS (95% CI)
≥9.4	24	16	79.2%	58.3%	35.4%	30.3%	2.4 years [1.7–2.9]	2.2 years [0.9–3.5]
<9.4	60	3	98.3%	94.5%	94.5%	94.5%	4.4 years [4.1–4.6]	—

**Table 3:** Overall survival (OS) rate of stages I and II melanoma patients according to MIA serum concentration.

MIA ng/mL	Number of patients	Number of deaths	1-year OS	2-year OS	3-year OS	4-year OS	Mean OS (95% CI)	Median OS (95% CI)
≥9.4	24	14	87.5%	62.5%	43.8%	38.9%	2.9 years [2.3–3.6]	2.9 years [1.7–4.0]
<9.4	60	3	98.3%	96.2%	93.3%	93.3%	4.4 years [4.1–4.6]	—

**Table 4:** Multivariate logistic regression of death risk for MIA cut-off value of 9.4 ng/mL and stages I and II patients.

Term	Odds ratio	95%	CI	Coefficient	S. E.	*Z*-statistic	*P* value
MIA ≥ 9.4 ng/mL	**33.1835**	7.2470	151.9457	3.5021	0.7763	4.5114	**0.0000**
TNM stage (I/II)	**7.4541**	1.2037	46.1601	2.0088	0.9303	2.1593	**0.0308**

## References

[1] Liu S, Kirschmeier P, Simon J, Seidel-Dugan C, Puhlmann M (2008). Prognostic and predictive molecular markers in cutaneous malignant melanoma: the first step toward personalized medicine. *Current Pharmacogenomics and Personalized Medicine*.

[2] Essner R, Lee JH, Wanek LA (2004). Contemporary surgical treatment of advanced-stage melanoma. *Archives of Surgery*.

[3] Balch CM, Gershenwald JE, Soong S (2009). Final version of 2009 AJCC melanoma staging and classification. *Journal of Clinical Oncology*.

[4] Mocellin S, Zavagno G, Nitti D (2008). The prognostic value of serum S100B in patients with cutaneous melanoma: a meta-analysis. *International Journal of Cancer*.

[5] Bosserhoff A (2005). Melanoma inhibitory activity (MIA): an important molecule in melanoma development and progression. *Pigment Cell Research*.

[6] Bosserhoff AK (2006). Mia1. *UCSD-Nature Molecule*.

[7] Bosserhoff A-K, Stoll R, Sleeman JP, Bataille F, Buettner R, Holak TA (2003). Active detachment involves inhibition of cell-matrix contacts of malignant melanoma cells by secretion of melanoma inhibitory activity. *Laboratory Investigation*.

[8] Kruijff S, Hoekstra HJ (2012). The current status of S-100B as a biomarker in melanoma. *European Journal of Surgical Oncology*.

[9] Al-Shaer M, Gollapudi D, Papageorgio C (2010). Melanoma biomarkers: vox clamantis in deserto (review). *Oncology Letters*.

[10] Bosserhoff A-K, Kaufmann M, Kaluza B (1997). Melanoma-inhibiting activity, a novel serum marker for progression of malignant melanoma. *Cancer Research*.

[11] Meral R, Duranyildiz D, Tas F (2001). Prognostic significance of melanoma inhibiting activity levels in malignant melanoma. *Melanoma Research*.

[12] Hofmann MA, Gussmann F, Fritsche A (2009). Diagnostic value of melanoma inhibitory activity serum marker in the follow-up of patients with stage I or II cutaneous melanoma. *Melanoma Research*.

[13] Essler M, Link A, Belloni B (2011). Prognostic value of [18F]-fluoro-deoxy-glucose PET/CT, S100 or MIA for assessment of cancer-associated mortality in patients with high risk melanoma. *PLoS ONE*.

[14] Hofmann MA, Schicke B, Fritsch A (2011). Impact of lymph node metastases on serum level of melanoma inhibitory activity in stage III melanoma patients. *Journal of Dermatology*.

[15] Auge JM, Molina R, Filella X (2005). S-100*β* and MIA in advanced melanoma in relation to prognostic factors. *Anticancer Research*.

